# Interactions of a Water-Soluble Glycofullerene with Glucose Transporter 1. Analysis of the Cellular Effects on a Pancreatic Tumor Model

**DOI:** 10.3390/nano11020513

**Published:** 2021-02-18

**Authors:** Edyta Barańska, Olga Wiecheć-Cudak, Monika Rak, Aleksandra Bienia, Anna Mrozek-Wilczkiewicz, Martyna Krzykawska-Serda, Maciej Serda

**Affiliations:** 1 Faculty of Biochemistry, Biophysics and Biotechnology, Jagiellonian University, 30-387 Kraków, Poland; edyta.anna.b@gmail.com (E.B.); olga.wiechec@doctoral.uj.edu.pl (O.W.-C.); monika.rak@uj.edu.pl (M.R.); aleksandra.bienia@student.uj.edu.pl (A.B.); 2 August Chełkowski Institute of Physics and Silesian Centre for Education and Interdisciplinary Research, University of Silesia in Katowice, 41-500 Chorzów, Poland; anna.mrozek-wilczkiewicz@us.edu.pl; 3 Institute of Chemistry, University of Silesia in Katowice, 40-006 Katowice, Poland

**Keywords:** [60]fullerene, pancreatic cancer, drug delivery vehicles, glycofullerenes

## Abstract

In recent years, carbon nanomaterials have been intensively investigated for their possible applications in biomedical studies, especially as drug delivery vehicles. Several surface modifications can modulate the unique molecular structure of [60]fullerene derivatives, as well as their physicochemical properties. For this reason, covalent modifications that would enable a greater water solubilization of the fullerene buckyball have been rapidly investigated. The most exciting applications of fullerene nanomaterials are as drug delivery vectors, photosensitizers in photodynamic therapy (PDT), astransfection or MRI contrast agents, antimicrobials and antioxidants. From these perspectives, the glucose derivatives of [60]fullerene seem to be an interesting carbon nanomaterial for biological studies. It is well-known that cancer cells are characterized by an increased glucose uptake and it has also been previously reported that the glucose transporters (GLUTs) are overexpressed in several types of cancers, which make them attractive molecular targets for many drugs. This study explored the use of a highly water-soluble glycofullerene (called *Sweet*-C_60_) in pancreatic cancer studies. Here, we describe the PANC-1 cell proliferation, migration, metabolic activity and glycolysis rate after incubations with different concentrations of *Sweet*-C_60_. The final results did not show any influence of the *Sweet*-C_60_ on various cancer cellular events and glycolysis, suggesting that synthesized glycofullerene is a promising drug delivery vehicle for treating pancreatic cancer.

## 1. Introduction

Recently, carbon nanomaterials have become increasingly popular in biological studies, as can be seen by the oncology and clinical reports [1,2]. Moreover, some of them have been officially approved for use in cancer treatment [3]. The [60]fullerene molecule (C_60_) has also become a subject of interest due to the plethora of synthetic modifications that increase its water solubility [4]. [60]fullerene is a spherical molecule that is shaped like a regular icosahedron and is composed of 60 carbon atoms. As the [60]fullerene molecule is insoluble in water, several synthetic approaches have been developed in the quest to increase its bioavailability. Attaching polar functional groups to the [60]fullerene molecule leads to increased solubility in aqueous media [5,6,7]. As described in our previous study, high water solubility for fullerene nanomaterials can be obtained by using the Bingel–Hirsch cyclopropanation reaction with designed malonic acid derivatives that have several hydroxy, amino or carboxyl groups [8,9,10]. It is also worth mentioning that [60]fullerenes can form lipid-like systems that are able to penetrate cell membranes thanks to their unique spherical structure, which has a strong non-polar character.

**Figure 1:**
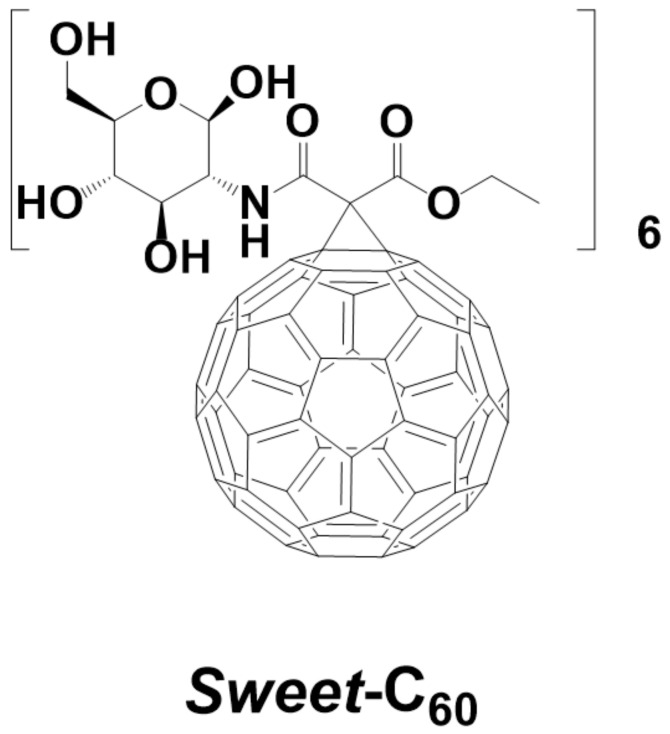
Chemical structure of the water-soluble glycofullerene *Sweet*-C_60_.

Moreover, the [60]fullerene derivatives can also be convenient scaffolds for delivering drugs to targeted cellular sites [11] to target the nuclear pore complex, as well as the tumor vasculature [12]. Other applications of buckyballs include photodynamic therapy (PDT), the transfection of DNA and siRNA, MRI contrast agents, antimicrobials and antioxidants [13,14,15]. Among others, [60]fullerene compounds with sugar functional groups, which are also called glycofullerenes, are attractive nanomaterials for pancreatic cancer studies due to their cellular localization inside the nuclear envelope, as well as their ability to generate reactive oxygen species after illumination with blue LEDs [8]. Our recent studies reported inhibitory activity for glycofullerenes on the non-receptor tyrosine kinases, mainly Fyn and BTK, which are promising targets for anti-cancer therapies [16]. Furthermore, it is well-known that cancer cells are characterized by an increased glucose uptake; hence, glucose-derived [60]fullerene nanomaterials may improve their location in cancer cells and thus increase the effectiveness of targeted therapy [17]. It has been reported that the glucose transporters (GLUTs) are overexpressed in several types of cancers, which makes them attractive molecular targets for many drugs [18]. Here, we present data that describe the cellular and molecular interactions of a photoactive [60]fullerene derivative (*Sweet*-C_60_ Figure 1) in the presence of the pancreatic cancer cells PANC-1. The synthesis of *Sweet*-C_60_ and its cellular localization in the pancreatic stellate cells has already been described in our previously published report [8]. This study explored the interactions of glycofullerene with the pancreatic cancer cells PANC-1. This type of tumor is characterized by having the worst prognosis [19] and it is refractory to treatment with high levels of mortality [20]. The average survival rate of patients with detected pancreatic cancer does not exceed six months and only 3–6% of patients survive for five years [21,22]. Unfortunately, the frequency and number of deaths that are caused by pancreatic tumors are gradually increasing [23]. The invasiveness and difficulty in treating pancreatic cancer are caused by many factors, such as mutations in the genes [24]; microRNA regulation disorders [25]; disorders in the signaling pathways, including apoptosis [26]; the presence of cancer stem cells [27]; epithelial–mesenchymal transition; hypoxia [28,29]; and increased angiogenesis [30,31].

Moreover, pancreatic cancer does not have any specific symptoms even after many years, which is why it is usually only detected at an advanced stage [19]. Although the research in recent years has contributed to a better understanding of genomic and transcriptomic pancreatic cancer [32], treating this cancer is still a challenge. Additionally, innovative and more effective treatment methods are still sorely needed, despite the advances in surgery, radiotherapy and chemotherapy. It seems that pancreatic cancer may be a justifiable target for the use of glycofullerene nanomaterials due to its increased glucose consumption, as well as the overexpression of the GLUTs. It has also been reported that anti-Glut-1 antibodies may disrupt the Glut-1 transporter functions and decrease tumor growth [33]. A high GLUT-1 expression was previously described in PANC-1 cells, which showed higher levels of Glut-1 mRNA and protein levels than normal pancreatic cells. The aforementioned experimental observation was verified using several biochemical assays as well as patient-derived cell lines and PET experiments using ^18^F-deoxyglucose [34]. This article presents a study of the effects of the fullerene nanomaterial *Sweet*-C_60_ on human pancreatic ductal adenocarcinoma (PANC-1). The results described here concern the cellular proliferation, glycolysis, migration and metabolic activity of PANC-1 cells after incubations with different concentrations of the [60]fullerene derivative. In addition, the effects of the fullerene nanomaterial on the glucose transporter (GLUT-1) expression were also studied. The study also attempted to determine the toxicity and multidirectional effects of *Sweet*-C_60_ on pancreatic cancer cells.

## 2. Materials and Methods

### 2.1. Cell Culture

In this study we used PANC-1, human pancreatic ductal adenocarcinoma (ATCC^®^ CRL-1469™), which was purchased from Sigma-Aldrich. The cell line was cultured at 37 °C, 5% CO_2_, in DMEM media (Sigma-Aldrich, St. Louis, MO, USA), which had been supplemented with 10% fetal bovine serum (Sigma-Aldrich, St. Louis, MO, USA) and penicillin (50 U/mL) and streptomycin at a concentration of 50 ng/mL (Polpharma, Poland).

### 2.2. Glycofullerene Synthesis and Application

The *Sweet*-C_60_ was synthesized using the previously described methodology and the fullerene nanomaterial was transferred through 0.45 µm membranes before being lyophilized for the further cellular experiments [8]. The *Sweet*-C_60_ was used at concentrations of 0.01 and 0.1 mg/mL. The solutions of the compound with various concentrations were prepared in the culture medium. Different incubation durations (in a range from 2 to 120 h) were used depending on the experimental conditions.

### 2.3. Cell Counting Using a Hemocytometer

The cell proliferation rate was estimated by cell counting using a Bürker’s hemocytometer (Heinz Herenz, Hamburg, Germany). The cells were seeded into six-well plates (60,000 cells per well) and cultured in standard conditions for 48 h. Afterward, the cells were treated with 0.01 or 0.1 mg/mL [60]fullerene derivative solution. The pancreatic cancer cells were then incubated with 0.25% trypsin (Sigma-Aldrich, St. Louis, MO, USA) for one minute. Next, the cells were removed from the suspension, diluted in a serum culture medium and counted using the hemocytometer. The cells were counted every 24 h for five days.

### 2.4. The MTT (3-(4,5-Dimethylthiazol-2-yl)-2,5-diphenyltetrazolium bromide) Assays

The metabolic activity of the cells was measured using MTT tetrazolium dye (3-(4,5-dimethylthiazol-2-yl)-2,5-diphenyltetrazolium bromide) (Sigma-Aldrich, St. Louis, MO, USA). The cells were seeded into 12-well plates (20,000 cells per well) and cultured in standard conditions for 48 h. The cells were then treated with 0.01 or 0.1 mg/mL of the glycofullerene *Sweet*-C_60_ solution. After 48 or 120 h of incubation, the cells were supplemented with an MTT stock solution (0.5 mg/mL) and incubated for 90 min. The formazan crystals were dissolved in methanol (Avantor, Poland) and DMSO (Sigma-Aldrich, St. Louis, MO, USA) solution (1:1). The absorbance was measured at a wavelength of 565 nm with an Infinite^TM^ 200 Quad-4 Monochromator^TM^ plate reader (Tecan, Switzerland). Three independent experiments were conducted.

### 2.5. Wound-Healing Assay

The migration rate of the tested cells was determined using a wound-healing assay. The cells were plated into six-well plates (200,000 or 50,000 cells per well) and cultured in standard conditions for 24 h. Afterwards, the cells were treated with 0.01 or 0.1 mg/mL of the [60]fullerene derivative solution. After 48 h (200,000 cells per well) or 120 h (50,000 cells per well) of incubation, a wound (scratch) was made using a sterile tip. Pictures of the wound were taken at set intervals until it was covered once again. The wound area was analyzed using ImageJ (Wayne Rasband, National Institute of Health, Bethesda, MD, USA).

### 2.6. Cell Migration

Time-lapse images of the PANC-1 cells migration were taken using a Leica DMI6000B microscope equipped with a Leica DFC360 FX CCD camera. The cells were plated into the wells of 24-well plates and incubated with 0.01 or 0.1 mg/mL of a *Sweet*-C_60_ solution for 12 or 36 h. On the day of the measurements, the cell density was 5 or 10%. Time-lapse images were taken for 12 h at five-minute intervals.

### 2.7. Western Blot

#### 2.7.1. Procedure for the GLUT-1 Protein

The GLUT-1 protein assay was performed 48 and 120 h after the cells had been incubated with 0.01 or 0.1 mg/mL of the [60]fullerene derivative solution. Cell lysis was performed on ice using a lysing buffer that contained 1 mM of protease inhibitor cocktail (Roche, Switzerland), 0.1 mM PMSF (Sigma-Aldrich, St. Louis, MO, USA), 1 mM sodium orthovanadate (Sigma-Aldrich, St. Louis, MO, USA), an RIPA buffer (containing nonylphenol ethoxylates, Thermo Scientific)) and SDS. The cells with the lysis buffer had been incubated on ice for 10 min and then frozen at −80 °C. The cells were then thawed on ice and centrifuged at 16,000 RPM for 10 min at 4 °C. The supernatant formed as a result of centrifugation was transferred to newly prepared 1.5 mL Eppendorf tubes, which were stored at −80 °C. The amount of protein was measured using Bicinchoninic Acid (BCA) (Sigma-Aldrich, St. Louis, MO, USA) and Copper II sulfate (Sigma-Aldrich, St. Louis, MO, USA). Proteins were run on Mini-Protecan^®^ gels (4–15% of polyacrylamide, Thermo Fisher Scientific, USA). The electrophoresis was carried out at an initial voltage of 90 V, which was increased to 100 V. The proteins were transferred onto a nitrocellulose, blocked with 5% non-fat milk in a TBS-T buffer for 1.5 h and incubated with the primary antibodies against GLUT-1 (ab115730, Abcam, Cambridge, UK) and vinculin (ab129002) (Abcam, Cambridge, UK) at 4 °C overnight. Then, the membrane was washed three times in TBS-T and incubated with the suitable secondary antibodies for 1.5 h. The signals were detected using chemiluminescence using a SuperSignal^TM^ West Pico PLUS Chemiluminescence Substrate (Thermo Fisher Scientific, Waltham, MA, USA). Quantitative protein analysis was performed using ImageJ (Wayne Rasband, National Institute of Health, Bethesda, MD, USA).

#### 2.7.2. Procedure for the HIF-1α, HO-1 and MMP-2 Proteins

The PANC-1 cell line was seeded in 3 cm Petri dishes (Nunc) at a density of 500,000 cells/well. After 24 h, 0 and 1 mg/mL solutions of *Sweet*-C_60_ at various concentrations were added and the cells were incubated for the next 24 h. Next, the cells were harvested via trypsinization, washed with PBS, centrifuged, suspended in a RIPA buffer containing a Halt Protease Inhibitor Cocktail (final concentration of 25×) and a Halt Phosphatase Inhibitor Cocktail (final concentration of 10×) along with 0.5 M EDTA (all from Thermo Scientific, USA) and lysed for 20 min on ice. Then, the lysates were sonicated, centrifuged at 10,000 rpm for 10 min at 4 °C, then the supernatants were collected. The protein concentration was determined using a Micro BCA™ Protein Assay Kit (Thermo Scientific, USA). Equal amounts of the proteins (20 µg) were electrophoresed on SDS-Page gels (10% polyacrylamide + 0.1% SDS). After the electrophoresis the proteins were transferred onto nitrocellulose membranes which were blocked in 5% non-fat milk (prepared in PBS containing 0.1% Tween-20 (TPBS)) for 1 h. After blocking, the membranes were incubated with specific primary antibodies—HIF-1α (D2U3T) Rabbit mAb #14179, HO-1 (D60G11) Rabbit mAb #5853, MMP-2 (D8N9Y) Rabbit mAb #13132 and GAPDH (14C10) Rabbit mAb #2118—overnight at 4 °C. The next day the membranes were washed and incubated with the horseradish peroxidase (HRP)-conjugated secondary antibodies (Anti-rabbit IgG, HRP-linked Antibody #7074) for 1 h at room temperature. The antibodies were purchased from Cell Signaling (Danvers, MA, USA). They were diluted 1:1000 in 5% milk in TPBS. Lastly, the membranes were washed and incubated with a SuperSignal™ West Pico Chemiluminescent Substrate (Thermo Scientific, Waltham, MA, USA). The chemiluminescence signals were captured using a ChemiDoc™ XRS+ System (Bio-rad, Hercules, CA, USA). The experiments were performed at least three times. The densitometric analysis was performed using ImageJ 1.41 software.

### 2.8. Glycolysis Assay (Extracellular Acidification)

Extracellular acidification was detected using a Glycolysis Assay kit (Abcam, UK). The cells were seeded into a 96-well black wall with clear flat bottom plates (30,000 cells per well) and cultured in standard conditions for 24 h, after which the cells were treated with 0.01 or 0.1 mg/mL of the *Sweet*-C_60_ solution. After 24 h, the cells were incubated with reagents according to the manufacturer’s recommendations (ab197244). A CLARIO star plate reader (BMG Labtech, Germany) was used to measure the extracellular acidification. The signals were collected at 90-s intervals for about 120 min using excitation and emission wavelengths of 340 and 615 nm, respectively.

### 2.9. Statistical Analysis

Statistical analysis was performed using Statistica (StatSoft. Inc., Tulsa, OK, USA). Each experiment was repeated at least twice and between three and eight replicates were performed in each of them. The final result was the mean value ± the standard error of the mean (SEM). To determine the reliability of the differences in the comparative studies, the values of given experiments were verified using a one-way analysis of variance (ANOVA). The confidence interval was 95%, α = 0.05. In the event that *p* <0.05, the Newman–Keuls test was carried out to determine which groups differed from each other.

## 3. Results and Discussion

### 3.1. The Changes in the Rate of Proliferation

**Figure 2:**
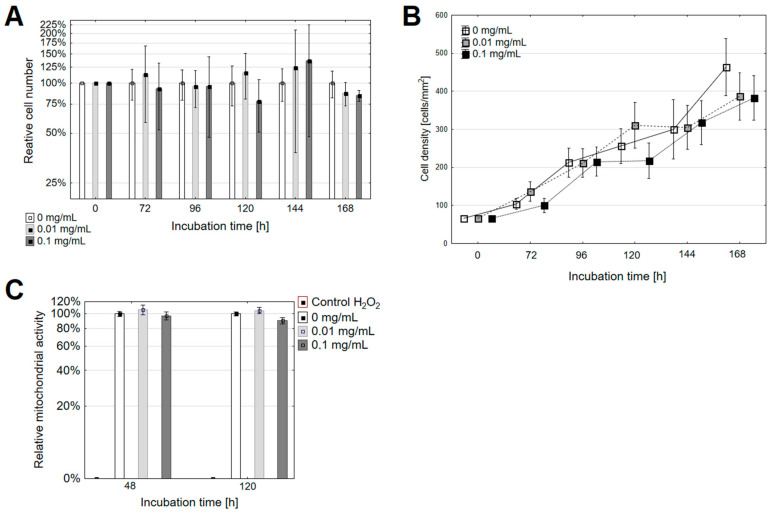
The changes in the (**A**,**B**) proliferation and (**C**) mitochondrial activity in the PANC-1 cells after they had been treated with different concentrations of the fullerene nanomaterial. Error bars are presented as (**A**,**B**) SD or (**C**) SEM. The relative cell number indicates the cell number in the experimental group at the correct time point relative to the untreated cell number at the same timepoint.

Based on the pioneering publications of Wilson et al., we supposed that the water-soluble [60]fullerene derivative might affect cellular pathways even though they are considered to be non-toxic without illumination [35,36,37]. Our previous studies showed that *Sweet*-C_60_ had a negligible in vitro toxicity even at high doses (1 mg/mL) and localized preferentially in the nucleus of the pancreatic stellate cells. Our cell proliferation study for five consecutive days showed no significant change in dose-dependent proliferation rate. The Kruskal–Wallis H (KW H) test results were *p* > 0.05 for all of the groups. By analyzing the selected long-term points as depicted in Figure 2, it was determined that incubating the PANC-1 cells with the glycofullerene led to a slightly slower proliferation rate (Figure 2B). It was found that after 72 h of incubation with the [60]fullerene derivative solution at a dose of 0.1 mg/mL, the relative number of PANC-1 cells was reduced to 77.8 ± 7.8% relative to the control (Figure 2A). The one-way ANOVA analysis was *p* = 0.014 relative to the control group. Next, a Newman–Keuls post hoc analysis was performed. A statistically significant difference was found between the 0.01 mg/mL group and the 0.1 mg/mL group (*p* = 0.011). Conversely, the incubation with *Sweet*-C_60_ for 120 h for both doses led to a decrease in the number of cells relative to the control (Figure 2A). Correspondingly, the number of cells after incubation with the glycofullerene at 0.01 mg/mL was 86.8 ± 4.1% compared to the control, while the relative number of cells after incubation at 0.1 mg/mL was 84.1 ± 1.8% of control. The one-way ANOVA analysis relative to the control group was *p* = 0.019. Statistically significant differences (Newman–Keuls post hoc analysis) were found between the control group and a concentration of 0.01 mg/mL (*p* = 0.026) and between the control group and a concentration of 0.1 mg/mL (*p* = 0.023). The results showed that after the 120-h incubation of the PANC-1 cells with the [60]fullerene derivative, the cell number/mm^2^ decreased as the concentration increased (Figure 2B). The results that were obtained after 24 h, 48 h and 96 h of incubation were not statistically significant. Moreover, the results showed that the time required for the PANC-1 cell division after the treatment with *Sweet*-C_60_ was slightly extended. The doubling time was 59.9 h for the untreated cells (control cells), 66.2 h for the low dose and 66.6 h for the high dose, respectively. The doubling times that were obtained were slightly higher than those presented in previous reports [38,39]. The obtained growth factors and cell doubling times showed that the cells that had been incubated with the highest (0.1 mg/mL) concentration of the [60]fullerene nanomaterial proliferated most slowly (growth factor = 0.01042), whereas the control cells proliferated the fastest (growth factor = 0.01156). The observed differences in the doubling time may have been due to the cell maintenance conditions, such as nutrient access, the surface area for growth and various stress factors. If there were significant differences in the cell proliferation in the study groups, one could suppose that glycofullerene nanomaterial has anti-tumoral cell division properties and is cytotoxic to them. Based on the current data, we do not support such a hypothesis.

Furthermore, the mitochondrial activity was investigated in the PANC-1 cells after their incubation with *Sweet*-C_60_ (Figure 2C). It was observed that treating the PANC-1 cells with *Sweet*-C_60_ at a dose of 0.01 mg/mL led to an insignificant increase in the metabolic activity. The number of metabolically active cells at a low dose of [60]fullerene derivative increased to 106.3 ± 7.7% relative to the control (incubation for 48 h) and 105.1 ± 5.2% (incubation for 120 h). In contrast, treating the PANC-1 cells with 0.1 mg/mL of *Sweet*-C_60_ led to a decrease in the metabolic activity relative to the control group. The relative metabolic activity was measured as 97.1 ± 6.2% (incubation for 48 h) and 90.2 ± 4.5% (incubation for 120 h). For both incubation durations, there were no statistically significant differences between the tested concentrations of the nanomaterial.

In the MTT metabolic test, the amount of formazan crystals formed was proportional to the oxidative activity of the mitochondria of the cells and to the number of metabolically active cells of a given population. It could therefore be concluded that the results obtained for the MTT test were analogous to the rate of cell proliferation. Both tests were consistent for the group of cells that had been treated with *Sweet*-C_60_ at a concentration of 0.1 mg/mL. At the selected time points, the proliferation rate of the treated cells was slower and the number of metabolically active cells was lower. However, for the concentration of 0.01 mg/mL, the number of metabolically active cells was higher in the control group. Additionally, the metabolic activity slightly decreased with the incubation duration at both concentrations. This phenomenon can be explained by the fact that, although the cells were still metabolically active, their proliferation was slightly slower at selected points over time. It can be assumed that if cells stop proliferating but are still metabolically active, they begin to metabolize glucose through the oxidative phosphorylation pathway [40]. Hence, glycofullerene treatment could inhibit the PANC-1 cell proliferation by changing the glucose metabolism pathways that are typical for the differentiated cells. On the other hand, it cannot be ruled out that a slight slowdown in metabolism and a slight slowdown in the proliferation over a long-term cell incubation might be caused by stress factors such as nutrient depletion.

### 3.2. The Migration Rate and Wound Healing

**Figure 3:**
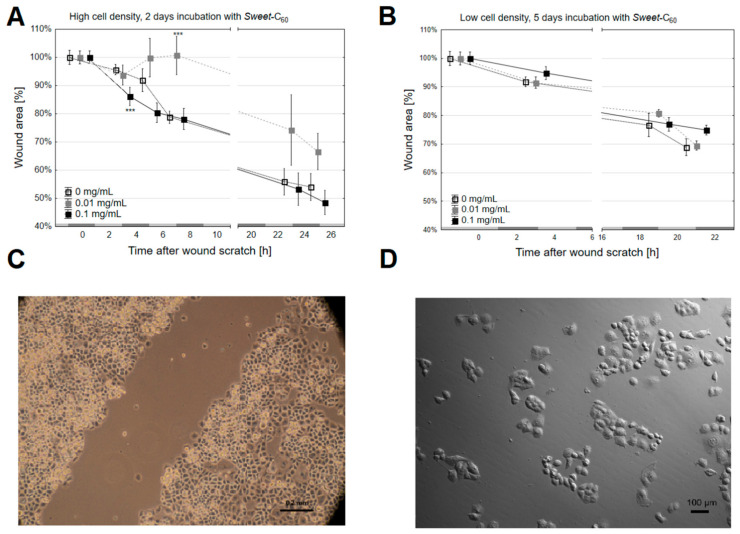
The wound-healing test was performed for the PANC-1 cells that had been incubated with *Sweet*-C_60_ for (**A**) 48 h and (**B**) 120 h. The cells were prepared at different densities in order to manage different incubation times: (**A**) a high cell density of 200,000 cells and (**B**) 50,000 cells. Error bars presented as SD. (**C**) A representative image of the wound scratch made in the PANC-1 colony; (**D**) a representative image of the PANC-1 cells that were used for a time-lapse movement analysis under a Leica microscope. The gray bars on the x-axis (**A**,**B**) indicate time periods.

The results depicted in Figure 3A–D were obtained for the wound-healing test and showed how a wound surface might decrease (100% was the surface at the time the wound was made) depending on the duration (x-axis). After 48 h of incubation with *Sweet*-C_60_, the changes in the rate of wound healing were observed between two and four hours of measurement (in the initial phase, the rate of migration was steady at all levels). It was shown that, in the last hour of measurement (25.5 h), the wound surface decreased to 54.1 ± 4.8% of its initial surface area in the control group, 66.6 ± 6.4% for the group that had been treated with *Sweet*-C_60_ at a concentration of 0.01 mg/mL and 48.5 ± 4.4% in the 0.1 mg/mL group. However, for the incubation for 120 h, in the last hour of measurement (22 h), the remaining wound area was 69 ± 3.0% for the non-treated group, 69.5 ± 1.6% for the 0.01 mg/mL group and 74.9 ± 1.0% for the 0.1 mg/mL group. Based on the determined trend lines and their equations, it was shown that, for both incubation durations (48 h and 72 h), the control group wound healed the fastest (untreated cells), while the slowest wound healed when cells had been treated with the *Sweet*-C_60_ at a dose of 0.01 mg/mL. The coefficient α for the linear decreasing function for the control group and doses of 0.01 mg/mL and 0.1 mg/mL of *Sweet*-C_60_ were −0.0182, −0.0141 and −0.0162, respectively (for 48-h incubation) and −0.0132, −0.0119 and −0.0120 (for 120-h incubation), respectively. To summarize, when the proliferation time was considered, the wound-healing assay did not provide a clear answer about the impact of *Sweet*-C_60_ on the pancreatic cancer cell migration. It has been postulated by some authors that PANC-1 cells have motility and they appear to migrate mainly as single cells, which is why a time-lapse migration analysis was performed in the next step [39]. Here, it was clearly observed that the PANC-1 cells had a very limited migration. Both the cells in the control group and the cells that had been incubated with *Sweet*-C_60_ (different concentrations and incubation durations) did not show any significant movement, displacement or polarity in any of the tested conditions. The representative data is presented in Supplementary Movie S1 (PANC-1 control) and Movie S2 (PANC-1 that was treated with *Sweet*-C_60_). 

Moreover, it has also been postulated that glucose concentration might be an important factor for cancer cell migration [41]. That is why the observation that *Sweet*-C_60_ did not influence cell mobility is noteworthy, as it makes it a promising nanoscale drug delivery tool for anti-cancer studies

### 3.3. Expression of the Glucose Transporter Protein (GLUT-1)

**Figure 4:**
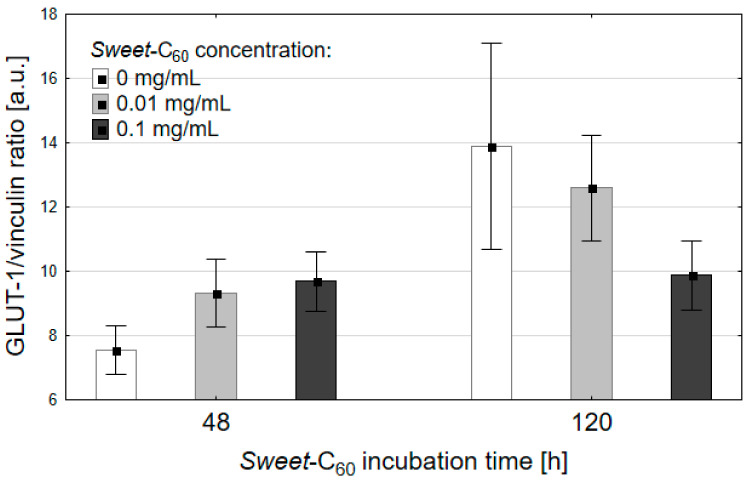
The effects of the fullerene nanomaterial *Sweet*-C_60_ on the activity of GLUT-1 protein. Error bars are presented as SD.

Expression of the GLUT-1 protein in the PANC-1 cells was investigated using the Western blot technique and is represented as its ratio to vinculin (the reference protein, Figure 4). The data is represented as the mean± SEM, n = 3. The cancer cells were incubated in a media with a different concentrations of the fullerene nanomaterial and with no [60]fullerene as the control. The cells were incubated in standard conditions for 48 and 120 h without changing the media. Due to the increased number of cells and decreasing nutrient supply during the incubation time, the expression of GLUT-1 significantly increased over time, especially in the control group (*p* = 0.0495, Kruskal–Wallis test). Interestingly, the level of the GLUT-1 protein did not change significantly during the incubation with *Sweet*-C_60_. At the 48th hour after the incubation with the fullerene nanomaterial, there was a slight increase of GLUT-1 for both of the *Sweet*-C_60_ concentrations—this effect could be explained by the increased possibility for the cells to uptake additional glucose. What is more, after 120 h of incubation, there was a slight increase of GLUT-1 protein for the 0.01 mg/mL concentration, but there was no change of GLUT-1 in the cells that had been incubated with the 0.1 mg/mL concentration of the glycofullerene. These results suggest that a high concentration of *Sweet*-C_60_ in cells that were grown in a medium did not induce cancer cells to express high GLUT-1 concentration over the incubation period.

### 3.4. Expression of the Proteins Related to Cancer Cell Migration

The cancer nanotechnology literature includes descriptions of several interactions between different types of water-soluble [60]fullerenes (cationic, neutral or anionic) that have enzymes that could be crucial for anti-cancer studies. We focused here on three different proteins, MMP-2, HIF-1 and HO-1, which have been previously reported and could be inhibited/activated after the cancer cells had been incubated with different fullerene nanomaterials [42,43]. The influences of the studied fullerene nanomaterial on the MMP-2, HIF-1 and HO-1 proteins were studied using the Western blot technique after 24 and 72 h of incubation (Supporting Information, Figure S1). There were no significant changes to the studied proteins in the tested conditions. This observation indicates that the tested compound could be used as a convenient vehicle for drug delivery for treating pancreatic tumors.

### 3.5. Glycolysis Assay

**Figure 5:**
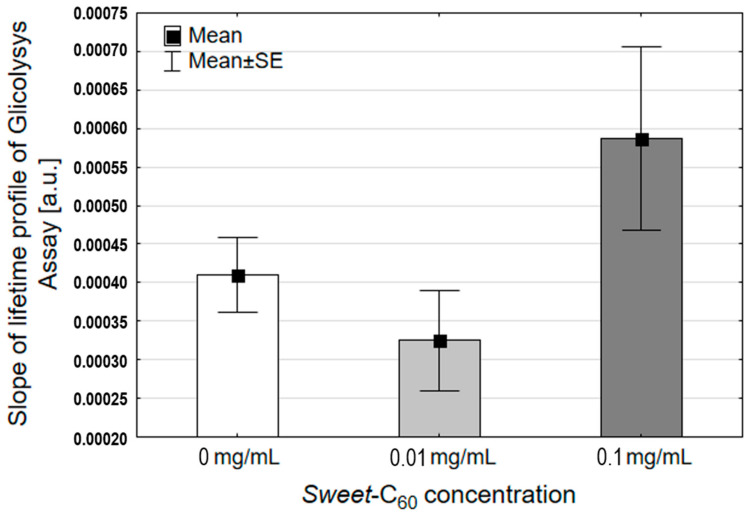
The effects of water-soluble glycofullerene *Sweet*-C_60_ on glycolysis in the PANC-1 cells. Error bars are presented as SD.

GLUT-1 is a well-known protein that is involved in regulating the cell glycolysis cycle, which has been demonstrated on cancer cell lines such as prostate and breast tumors [44]. Moreover, an enhanced glycolysis (Warburg effect) is one of cancer’s hallmarks; it is associated with extremely aggressive cancers and is used for developing targeted cancer nanotechnologies [45]. For example, cancer cells in the hypoxic areas often switch their metabolism to the glycolysis pathway. Our previous report described the effects of *Sweet*-C_60_ on the OH-1 levels and also indicated that the induction of autophagy took place after incubating the PANC-1 cells with *Sweet*-C_60_ [16]. The autophagy process has been described as being connected with glycolysis in pancreatic cancer [46]. Here we observed a slight increase in the glycolysis rates (Figure 5), which were measured by the extracellular acidification rate (ECAR) for the higher concentration of glycofullerene (0.1 mg/mL (*p* = 0.08)). These results suggest that while additional glucose availability might be related to an enhanced glycolysis, the possible effect is not strong and should not have any influence on migration of cells.

## 4. Conclusions

The results showed that higher doses of the fullerene nanomaterial *Sweet*-C_60_ do not have a significant influence on cell proliferation, metabolic activity or migration potential. Although a slight increase in the glycolysis process is possible for the higher concentrations of *Sweet*-C_60_, based on all of the presented results, it does not cause an increased migration of pancreatic cancer cells. Moreover, the presented data indicates that *Sweet*-C_60_ could be a promising drug delivery vehicle due to its lack of influence on cell migration, proliferation and metabolism while, at the same time, as is presented in our previous study, accumulating in the PANC-1 cells. What is also important is that the lower doses of this nanomaterial led to a decrease in the rate of proliferation that was accompanied by an increase in their metabolic activity. This effect may indicate an inhibition of the proliferation as a result of the changes in the glucose metabolism due to cell differentiation. Moreover, the overexpression of the GLUT-1 protein after incubation with *Sweet*-C_60_ was also demonstrated. This effect may lead to sensitizing the cells to targeted therapies due to an increased glucose demand. The final results suggest potential applications of water-soluble [60]fullerenes in biomedical research, specifically in cancer nanotechnology.

## Data Availability

The data presented in this study are available on request from the corresponding author.

## Supplementary Materials

The following are available online at https://www.mdpi.com/2079-4991/11/2/513/s1, Figure S1: The relative concentration of selected proteins (MMP-2, HIF-1 and HO-1) measured by Western Blot technique; Video S1: PANC-1 cells movements (no treatment) and Video S2: PANC-1 cells movements after the treatment with *Sweet*-C_60_.
