# Supplementary material for: Interactions of a Water-Soluble Glycofullerene with Glucose Transporter 1. Analysis of the Cellular Effects on a Pancreatic Tumor Model
Source: Nanomaterials (Basel). 2021 Feb 18;11(2):513. doi: 10.3390/nano11020513 (PMC7922475; doi:10.3390/nano11020513)
Supplement: Supplementary file 1 [file nanomaterials-11-00513-s001.zip › supplementary files/supporting information.pdf]

A WATER-SOLUBLE GLYCOFULLERENE INTERACTIONS WITH GLUCOSE TRANSPORTER 1. ANALYSIS OF THE CELLULAR EFFECTS ON PANCREATIC TUMOR MODEL

Edyta Barańska[1], Olga Wiecheć-Cudak[1], Monika Rak[1], Aleksandra Bienia[1], Anna Mrozek-Wilczkiewicz[2], Martyna Krzykawska-Serda*[1] and Maciej Serda[3]

[1]*Faculty of Biochemistry, Biophysics and Biotechnology, Jagiellonian University, Kraków, Poland*

[2]*A. Chełkowski Institute of Physics and Silesian Centre for Education and Interdisciplinary Research, University of Silesia in Katowice, Chorzów, Poland*

[3]*Institute of Chemistry, University of Silesia in Katowice, Katowice, Poland*

*email: maciej.serda@us.edu.pl; martyna.krzykawska@uj.edu.pl

WESTERN BLOTS FOR MMP-2, HIF-1 and HO-1 proteins

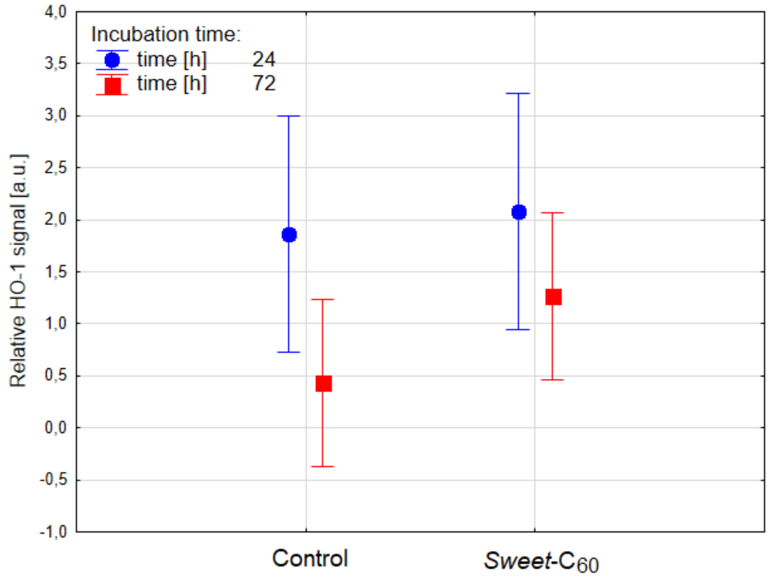
Incubation time:
time [h] 24
time [h] 72
Relative HO-1 signal [a.u.]
4,0
3,5
3,0
2,5
2,0
1,5
1,0
0,5
0,0
-0,5
-1,0
Control
Sweet-C_{60}

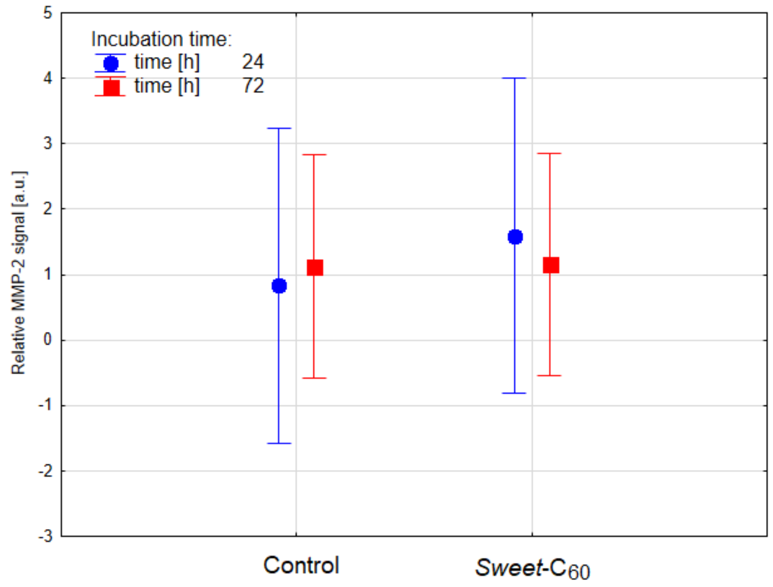
Incubation time:
time [h] 24
time [h] 72
Relative MMP-2 signal [a.u.]
5
4
3
2
1
0
-1
-2
-3
Control
Sweet-C_{60}

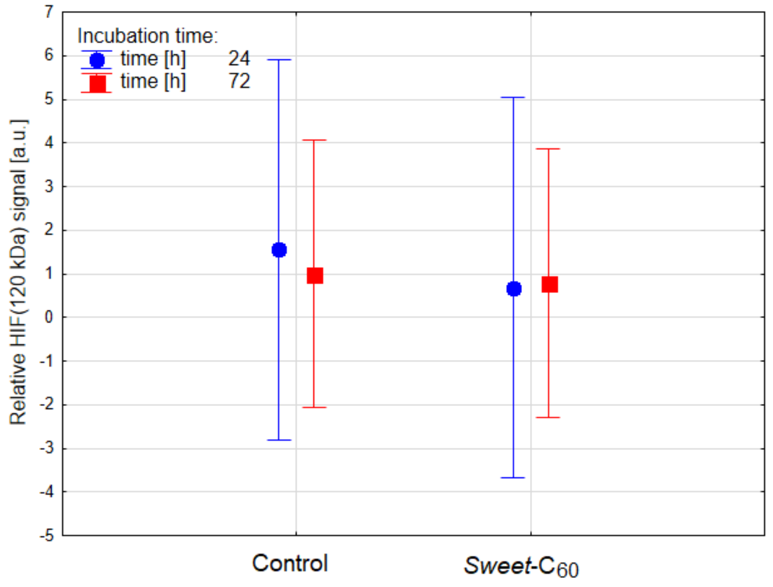
Incubation time:
time [h] 24
time [h] 72
Relative HIF(120 kDa) signal [a.u.]
7
6
5
4
3
2
1
0
-1
-2
-3
-4
-5
Control
Sweet-C_{60}

Figure S1

The relative concentration of selected proteins (MMP-2, HIF-1 and HO-1) measured by Western Blot technique. Data points presents signal intensity divided by signal intensity from reference protein (GAPDH), vertical bars denote 95 % confidence intervals. The Panc-1 cells were incubated with Sweet-C60 for 24 or 72 hours at concentration 0 and 1 mg/mL.
